# Facilitators and Barriers in the Implementation of a Digital Surveillance and Outbreak Response System in Ghana Before and During the COVID-19 Pandemic: Qualitative Analysis of Stakeholder Interviews

**DOI:** 10.2196/45715

**Published:** 2023-10-20

**Authors:** Basil Benduri Kaburi, Kaspar Wyss, Ernest Kenu, Franklin Asiedu-Bekoe, Anja M Hauri, Dennis Odai Laryea, Carolina J Klett-Tammen, Frédéric Leone, Christin Walter, Gérard Krause

**Affiliations:** 1 Department of Epidemiology, Helmholtz Centre for Infection Research Braunschweig Germany; 2 PhD Programme Epidemiology, Braunschweig-Hannover Braunschweig Germany; 3 Hannover Medical School Hannover Germany; 4 Swiss Tropical and Public Health Institute Allschwil Switzerland; 5 University of Basel Basel Switzerland; 6 Ghana Field Epidemiology and Laboratory Training Programme, University of Ghana Accra Ghana; 7 Public Health Division, Ghana Health Service Accra Ghana; 8 German Center for Infection Research Braunschweig Germany

**Keywords:** implementation, Surveillance Outbreak Response Management and Analysis System, SORMAS, barriers, facilitators, digital disease surveillance, outbreak response, COVID-19, pandemic, Ghana, mobile phone

## Abstract

**Background:**

In the past 2 decades, many countries have recognized the use of electronic systems for disease surveillance and outbreak response as an important strategy for disease control and prevention. In low- and middle-income countries, the adoption of these electronic systems remains a priority and has attracted the support of global health players. However, the successful implementation and institutionalization of electronic systems in low- and middle-income countries have been challenged by the local capacity to absorb technologies, decisiveness and strength of leadership, implementation costs, workforce attitudes toward innovation, and organizational factors. In November 2019, Ghana piloted the Surveillance Outbreak Response Management and Analysis System (SORMAS) for routine surveillance and subsequently used it for the national COVID-19 response.

**Objective:**

This study aims to identify the facilitators of and barriers to the sustainable implementation and operation of SORMAS in Ghana.

**Methods:**

Between November 2021 and March 2022, we conducted a qualitative study among 22 resource persons representing different stakeholders involved in the implementation of SORMAS in Ghana. We interviewed study participants via telephone using in-depth interview guides developed consistent with the model of diffusion of innovations in health service organizations. We transcribed the interviews verbatim and performed independent validation of transcripts and pseudonymization. We performed deductive coding using 7 a priori categories: innovation, adopting health system, adoption and assimilation, diffusion and dissemination, outer context, institutionalization, and linkages among the aspects of implementation. We used MAXQDA Analytics Pro for transcription, coding, and analysis.

**Results:**

The facilitators of SORMAS implementation included its coherent design consistent with the Integrated Disease Surveillance and Response system, adaptability to evolving local needs, relative advantages for task performance (eg, real-time reporting, generation of case-base data, improved data quality, mobile offline capability, and integration of laboratory procedures), intrinsic motivation of users, and a smartphone-savvy workforce. Other facilitators were its alignment with health system goals, dedicated national leadership, political endorsement, availability of in-country IT capacities, and financial and technical support from inventors and international development partners. The main barriers were unstable technical interoperability between SORMAS and existing health information systems, reliance on a private IT company for data hosting, unreliable internet connectivity, unstable national power supply, inadequate numbers and poor quality of data collection devices, and substantial dependence on external funding.

**Conclusions:**

The facilitators of and barriers to SORMAS implementation are multiple and interdependent. Important success conditions for implementation include enhanced scope and efficiency of task performance, strong technical and political stewardship, and a self-motivated workforce. Inadequate funding, limited IT infrastructure, and lack of software development expertise are mutually reinforcing barriers to implementation and progress to country ownership. Some barriers are external, relate to the overall national infrastructural development, and are not amenable even to unlimited project funding.

## Introduction

### Background

In the advent of affordable and robust IT tools in the 1990s, high-income countries began to switch from paper-based to electronic disease surveillance [1]. The pace of digitalization in public health surveillance and outbreak response has since gained momentum and spread to low- and middle-income countries (LMICs) [2]. This transition is, in part, a result of digital penetration, consolidated by the demonstrated utility of digital tools in public health practice [3-5]. Well-established electronic disease surveillance systems have many benefits. Some of these benefits are improved data quality (completeness, detail, and validity), timeliness of reporting, data standardization, automation of data analysis and visualization, prompt identification of public health events, and timely information sharing for public health action [6,7]. Thus, the benefits of digitalization in public health practice are many but so are the challenges. Frequently reported challenges of adopting electronic health systems include the technical challenges of technology, absence of basic necessary infrastructure such as electricity, implementation costs, workforce capacities and attitudes, and organizational factors [8-10]. These challenges are dynamic and vary in scope and complexity across geopolitical, socioeconomic, cultural, and organizational settings [10-12]. Therefore, some electronic systems have not made it beyond pilots to sustained nationwide institutionalization, especially in LMICs [10,13-18].

The cross-border geographical spread of outbreaks and the multidisciplinary response requirements made it more apparent than ever that conventional paper-based systems were simply inadequate to keep pace with the evolution of outbreaks [3,19-21]. Furthermore, most digital systems are designed for 1 or a limited set of response tasks, such as contact tracing, case management, data collection and transmission, data collation and analysis, communication, and coordination [22]. These limitations require public health systems to use multiple systems to accomplish the complete set of public health emergency response tasks—a detraction from the expected workload alleviation and efficiency of task performance. These challenges notwithstanding, the introduction of digital innovations for surveillance in LMICs has typically been welcomed by public health actors to bridge the service delivery gaps for which such innovations hold great promise. In particular, the occurrence of recent major outbreaks, namely, the West African Ebola Virus Disease outbreak and the COVID-19 pandemic, spurred an astounding transformation of electronic public health surveillance and outbreak response tools in Africa—one of which is the Surveillance Outbreak Response Management and Analysis System (SORMAS) [20,23-27].

The 2014 to 2016 West African Ebola Virus Disease outbreak motivated the invention of SORMAS. Beyond coordination, SORMAS integrates the business process management of disease control measures with the surveillance and detection of epidemics within 1 tool. However, compatibility with the existing Integrated Disease Surveillance and Response (IDSR) system of the World Health Organization (WHO)–Africa region was a prerequisite for adoption to maintain established work processes and offer a possibility of regional integration [28]. SORMAS is an open-source, mobile eHealth system. It is accessed via a web version by administrators and supervisors and via a mobile app version on tablets by field users. There exists a bidirectional communication between the web and mobile app versions for data synchronization. Functionally, it is a laboratory-integrated, case-based management system for routine surveillance and overall coordination of public health emergencies. It has disease process modules for notifiable diseases and nonspecific modules that are easily adaptable for emerging infectious diseases. The workflow is organized into interactive directories for the management of tasks, cases, contacts, events, laboratory samples, immunizations, points of entry, campaigns, statistics, reports, and users. SORMAS supports both indicator-based and event-based surveillance, real-time epidemiological analysis and data visualization, field coordination and response process management, bidirectional data transfer, and mobile offline functionality. It attained the global good maturity 4 years after its first deployment in Nigeria [23,26,29], where it was subsequently deployed for mpox (monkey pox) and other concurrent disease outbreaks [30,31].

In November 2019, the Ghana Health Service (GHS) started the pilot implementation of SORMAS for routine surveillance [31]. Then, 4 months into the pilot implementation phase, the COVID-19 pandemic spread to Ghana. As a consequence, the planned pilot implementation and progress evaluations that were aimed to improve strategies for a stepwise nationwide scale-up were interrupted. Nonetheless, given its demonstrated utility in the early phase of the pilot, Ghana adopted SORMAS for the COVID-19 pandemic response and, further, as the national eSurveillance and outbreak response management system for all its notifiable diseases. Thus, apart from the anticipated challenges of the implementation of digital innovations in normal times, the opportunity for a rapid nationwide scale-up of SORMAS during pandemic came with additional challenges.

### Objectives

Our study aimed to identify the facilitators of and barriers to the sustainable implementation of SORMAS in Ghana from the perspectives of stakeholders.

## Methods

### Study Design

We conducted a qualitative study among resource persons involved in the use and management of SORMAS in Ghana. These individuals represented GHS at the district, regional, and national levels; teaching hospitals; research laboratories; public health experts; and software developers for SORMAS (Table 1). We conducted the study in 16 districts across 2 administrative regions between November 2021 and March 2022. We purposively selected the Greater Accra and Ashanti regions for being the 2 most populous regions and COVID-19 epicenters of Ghana, where the use of SORMAS was most intensive. The Greater Accra Region was 1 of 2 pilot regions for SORMAS before the COVID-19 pandemic (2019), whereas the Ashanti Region started using SORMAS when the pandemic spread to Ghana.

We adapted the model of diffusion of innovations in health service organizations by Greenhalgh et al [32] to assess the implementation process. The model describes the dynamic interactions of factors, namely, the nature and value of the innovation, relevant health system antecedents and readiness for the innovation, method of adoption and assimilation, process diffusion and dissemination, implementation and consequences thereof, and relevant external influences on implementation and sustainability. Thus, the model provides a comprehensive and systematic approach to evaluating all aspects of the implementation of SORMAS from the innovation, the health system adopting it, its users, and the influence of local and international partners. We used these constructs to identify stakeholder perspectives about the barriers, facilitators, and sustainability strategies for system integration and institutionalization of SORMAS in Ghana.

**Table 1:** Categories and roles of resource persons involved in the implementation of Surveillance Outbreak Response Management and Analysis System (SORMAS) in Ghana (2022).

Category of study participant	Role in SORMAS implementation
National public health officers—GHS^a^	Decision-making Advocacy (public and private sectors and international) Administration and resource management Overall national leadership
Software developers	Feature development Release testing Debugging Server management (data hosting)
Regional surveillance supervisors	Workforce training and supervision Collection of user issues Feature requests and specifications Advocacy (regional-level and district-level stakeholders)
Frontline field users (district surveillance, contact, and health information officers)	Case identification and reporting Case investigation Contact tracing Sample collection and transportation Data entry and transmission Peer-to-peer training
Laboratory supervisors	Laboratory user training Laboratory data management Issue reporting to surveillance supervisors Coordination between surveillance units and laboratories
Laboratory officers	Sample management Data entry and validation Sample testing and reporting
Public health experts (workforce training institutions)	Participation in stakeholder meetings Participation in training and field deployments Advising decision makers about overall implementation

^a^GHS: Ghana Health Service.

### Context of SORMAS Implementation in Ghana

The *Helmholtz-Zentrum für infektionsforschung* (HZI; the Helmholtz Centre for Infection Research) Braunschweig–Germany, in collaboration with the African Field Epidemiology Network and the Nigeria Centre for Disease Control, developed and piloted the first version of SORMAS in 2015. With funding from the *Deutsche gesellschaft für internationale Zusammenarbeit* (GIZ; German Corporation for International Cooperation), HZI adapted the open-source version of SORMAS in 2016. On the basis of specifications of multinational team of epidemiologists and IDSR experts, the software developers integrated all change requests and lessons learned from the pilot to release an upgraded and first open version [31].

In 2017, a multistakeholder workshop was conducted in Ghana to consider the suitability of SORMAS as a national eSurveillance and outbreak response tool. Following a decision in 2018 to adopt SORMAS, a tripartite public-private partnership business model was adopted. In this model, GHS was the implementing public institution, Ghana Community Network System provided in-country IT service, and HZI was responsible for SORMAS development and maintenance. These 3 partners signed a memorandum of understanding, and the pilot implementation started in November 2019 in the Greater Accra and Upper West regions of Ghana. On January 29, 2020—a day before WHO declared COVID-19 as a public health emergency of international concern, HZI rolled out a COVID-19–specific module [33,34]. Ghana adopted this module and subsequently adapted it based on national response needs.

### Study Participants

**Figure 1:**
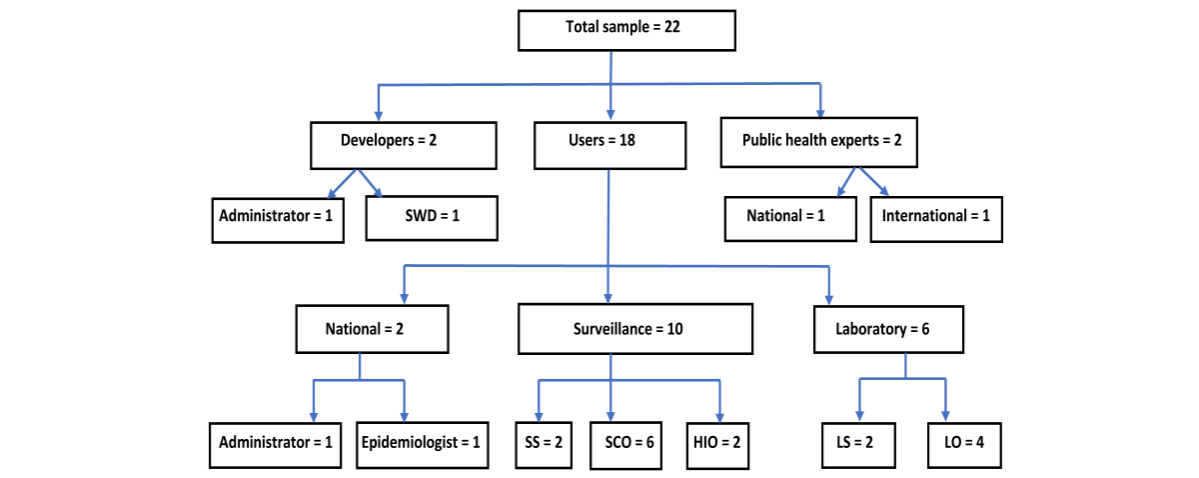
Distribution of study participants based on professional category. HIO: health information officer; LO: laboratory officer; LS: laboratory supervisor; SCO: surveillance and contact officer; SS: surveillance supervisor; SWD: software developer.

We recruited 22 study participants, distributed across the abovementioned 7 broad professional categories. These included officers at the national, regional, and district surveillance units and COVID-19–testing laboratories (Figure 1). All the users of SORMAS in the 2 study regions were invited to participate. We randomly selected equal numbers of frontline users based on region and distributed them equally between the urban and rural districts within each region. We purposively selected national officers, surveillance supervisors, and software developers based on their leading roles in implementation.

### Data Collection and Management

We designed a reference in-depth interview guide based on the model by Greenhalgh et al [32] such that some questions were the same for all study participants, and others were specific to their professional groups to match their roles and responsibilities in the implementation. We piloted the interview guides with a pretest for surveillance supervisors and frontline users in the Upper West Region, which had a mix of users from the pilot phase and users recruited for the pandemic response. Following this pilot, we revised the interview guides to improve their completeness in scope and the clarity of questions. We did not pilot the interview guides for the remaining 3 professional groups as their availability was limited by their roles and numbers. However, the research team discussed and reached consensus on the usability and soundness of their interview guides.

For each region, we grouped the frontline users who had previously given written informed consent based on the classification of their district of work as rural or urban. Each list was assigned random numbers generated using Excel (Microsoft Corporation). BBK then called the users from each list in ascending order of the magnitude of their assigned random numbers. He conducted the interviews via a direct telephone line and audio recorded them. He called each user twice within a span of 30 minutes by appointments. If there was no response, he called the next user in line from the list.

We transcribed the interviews verbatim with time stamps using MAXQDA Analytic Pro developed by Verbi GmbH. A research assistant listened to the audio recordings of all the interviews, validated the accuracy of the transcripts, and performed spell checks while maintaining the verbatim transcription. We performed proofreading and pseudonymization of the transcripts. We imported the validated and pseudonymized transcripts back into MAXQDA Analytic Pro (version 22.2.1) for analysis.

### Data Analysis

We coded the data deductively with a priori parent codes and subcodes consistent with the constructs of the model by Greenhalgh et al [32]. Our approach for coding was flexible to include emerging subcodes that related to some parent codes but were not explicitly outlined in the model. We assigned descriptive memos to all subcodes consistent with the constructs of the model, to ensure consistency of coding. BBK and FL performed the coding separately. CJK-T reviewed the coding performed by BBK and FL. BBK, FL, CW, and CJK-T compared and discussed the differences, built consensus where possible, and merged codes where the data did not make a practical difference to keep them separate. For example, we merged adoption and assimilation, diffusion and dissemination of the innovation, and system antecedents and system readiness for adopting the innovation to generate 1 composite parent code each. We also included access to the system, motivation, and trust as subcodes of diffusion and dissemination. We calculated the crude percentage agreements and Cohen κ coefficients at 95% confidence level to estimate the intercoder reliability for 7 final parent codes (Multimedia Appendix 1). We used the guidelines by Landis and Kock [35] to interpret intercoder agreement as follows: poor (κ≤0), slight (κ=0.01-0.20), fair (κ=0.21-0.40), moderate (κ=0.41-0.60), substantial (κ=0.61-0.80), and almost perfect (κ=0.81-1). We exported the coded segments as Excel sheets. We paraphrased the coded segments to obtain descriptive summaries. Next, we performed interpretative analysis on the descriptive summaries to identify key messages related to barriers and facilitators and how they influenced the implementation of SORMAS. We identified user recommendations for sustainability of implementation and categorized them according to their targeted stakeholders. GK reviewed and challenged the exhaustiveness of some of the interpretations we assigned to segments of the transcripts. We discussed further and refined the clarity and scope of some interpretations.

### Ethical Considerations

The study was approved by the GHS Ethics Committee (GHS-ERC: 016/03/21) and the Ethics Committee of the Hannover Medical School (Nr.9514_BO_S_2020). Through email correspondences and support of local coinvestigators, we obtained written informed consent (scanned copies) from all participants for interviews, audio recording, and publication of findings. We pseudonymized the data before analysis and ensured overall data protection, consistent with existing institutional and European General Data Protection Regulations—the basis of an approved data protection protocol for the study.

## Results

### General Results

Our study participants represented 7 categories of resource persons involved in the implementation of SORMAS in Ghana—73% (16/22) of whom were men. The median duration of the interviews was 59 (range 37-175) minutes. The intercoder reliability was almost perfect for adoption and assimilation (crude agreement=92.9%; κ=0.86, 95% CI 0.76-0.96), and substantial for the other 6 codes (crude agreement range 80%-87.5%; κ range 0.62-0.75; Multimedia Appendix 1).

### Facilitators and Barriers

#### Overview

We considered the barriers to and facilitators of implementation under 7 broad themes, each organized into subthemes consistent with the adapted model of diffusion of innovations in health service organizations (Multimedia Appendix 2). Overall, the identified barriers to and facilitators of implementation were similar across the 2 regions. The main facilitators resulting from the COVID-19 pandemic included the political support at the level of the presidency for the national scale-up, creation of Ghana-specific SORMAS development branch for national pandemic response needs, introduction of sample barcodes and scanners to speed up the workflow, and adoption of SORMAS by non-GHS facilities including private and state-owned research laboratories. In contrast, the main barriers to implementation occasioned by the pandemic included reduced quality of user trainings, shortage of tablets and other logistics arising from increased demand, and truncation of planned evaluation of the pilot implementation (Multimedia Appendix 2).

#### Innovation

The innovation referred to the technical aspects and work process of the object of implementation—SORMAS. The study participants identified barriers and facilitators that related to 6 subthemes, namely, suitability for task performance, nature of knowledge required for use, organizational compatibility, level of reinvention, trialability, and availability of technical support.

Compared with the conventional paper-based system, users named several relative advantages that SORMAS brings to their routine practice as managers, supervisors, and frontline field officers. These included its design for case-base data collection, real-time reporting, offline data collection capability of the mobile app, efficiency of work enabled by the integration of laboratory and surveillance procedures via the laboratory module, inbuilt data validation checks, and uniformity of data collection and reporting templates across the country each time the software is updated. In particular, the public health experts described the new possibility to generate and test hypotheses using the case-based data as the solution to a long-standing challenge in making optimum use of the data for research and project evaluations.

Barriers to the implementation of the innovation included user right restrictions that limit the scope of duties of supervisors, occasional slow system performance, and synchronization failures at peak data collection times. The national administrators and supervisors deemed the unavailability of an offline mode of the web version as a barrier to their work, especially when they are out in the field with poor internet connectivity.

Regarding how SORMAS compares with existing health information systems for task performance, a study participant explained that the innovations of SORMAS confer many relative advantages that made it easy for users to accept but also expressed concern about how unstable interoperability with existing systems could limit its expected benefits:

The tools that were being used for surveillance purposes – and of course, there was resistance to change like every normal...emmmm. When a new thing comes, there is that kind of thing. But I think that, the SORMAS came to complement the gaps that were already in existence because after several years of using these tools, there were gaps identified. And once SORMAS came in handy and was largely addressing most of the gaps that previously had been identified, the decision and getting people to use SORMAS did not become an issue. The other thing was that, if we were able to link the other soft-wares with SORMAS, we could allow data sharing with some of the soft-wares that we were already using. But like I said, there have been some challenges but they are being addressed.

PH_EX_NAT_42_QA_PT_240712_bka_ pan; Pos. 37

#### The Health System Antecedents and Readiness for the Innovation

System antecedents and readiness considered approaches to the conduct of surveillances and outbreak responses of GHS before the adoption of SORMAS. It also considered how its existing structures, resources, and work culture influenced the implementation of the innovation.

Study participants considered the introduction of SORMAS timely for 3 main reasons. First, there were advanced plans about transitioning from computer-assisted and paper-based IDSR system to an electronic IDSR. Second, the design of SORMAS was based on the content and workflow of the IDSR version already in use. Third, SORMAS is open to adaptations to the national context. This was demonstrated during the initial evaluation for adoption and further successful adaptations following deployment.

They identified 2 major health system barriers. First, there were challenges with the stability of interoperability of SORMAS with the main existing health data management system—District Health Information System–version 2 (DHIS2) used for routine health service delivery monitoring. Second, GHS lacked an internal IT infrastructural capacity for data hosting. This necessitated partnership with a local IT company to kick-start the implementation, for which a study participant expressed relief:

I believe if we did not have [name of the company] IT company by then, it would have been a very big problem for us. I’m sure it would have been like a stillbirth.

NA_DS_ADM_51_QA_PT_220606 _bka_pan; Pos. 30

#### Adoption and Assimilation

In this category, the study participants identified factors that related to early use of SORMAS by individual staff (adoption) and by the organization as a whole (assimilation). They identified facilitators and barriers under 3 subthemes, namely, approach to decision-making, evaluation of the system before and after adoption, and efforts at sustaining implementation.

The study participants identified the active participation of local developers on an internet hosting service for software development and version control (GitHub), biweekly development and innovation meetings among the 3 implementing partners, and software upgrade review meetings as facilitators that sustained the initial pilot deployment. In particular, the field users stated that the ability of SORMAS to include their request boosted their confidence in the system and facilitated assimilation.

They named 2 main barriers to this phase of implementation. First, the diversity of electronic tools and, second, the mass trainings of users (up to approximately 200/cohort compared with approximately 30 before the COVID-19 pandemic). They explained that there was wide diversity of electronic data collection tools in public, private, and quasi-government health facilities, for which managers would prefer interoperability instead of a total switch to SORMAS. The mass trainings were occasioned by the need to boost the number of contact tracers for COVID-19 response. They reported that this approach of introducing the system to them was ineffective for lack of adequate hands-on practice before deployment to the field.

#### Diffusion and Dissemination

Diffusion and dissemination assessed the factors that influence the time span needed to administer SORMAS and spread its use among individual officers and at all levels of the health system after adoption and assimilation. All categories of study participants identified the mutually reinforcing approaches of training as the most important facilitator of the spread of SORMAS. These included formal in-person workshops using real data for hands-on practice, internet-based remote support from regional and national teams, and peer-to-peer trainings on the job. The familiarity of users with smartphone apps and adequate computer skills emerged as another facilitator of gaining speedy competence on the use of SORMAS. The overarching facilitator was the intrinsic motivation of frontline users. They stated that their motivation was sustained by the efficiency that SORMAS brought to their work routines and their trust in the vision of leadership to transform public health practice. The use of intuitive graphic illustrations as a training aid that described the unique advantages of SORMAS facilitated user understanding and diffusion of the system, as expressed by a frontline user and peer trainer:

As I’m speaking with you, a brochure given to me at training is in front of me and I’m still holding my SORMAS small handbook with me here. As I speak with you, all the connections: how the arrows and those things, SORMAS users, technical issues, inter role operations, SORMAS features – everything is in front of me here. I love SORMAS so much and I believe that those who have engineered it have done a very good job for the country. So, I’m ever ready whenever I’m called upon, I will be able to help.

GA_AS_DCS_03_QA_PT_220811_bka_pan; Pos. 216

In addition, frontline users were sufficiently self-motivated to support the implementation efforts by using their personal income to buy internet data bundles to access the system when there were challenges in supply from supervisors:

We buy our own data, with our own personal money.

GA_MT_DMA_72_PT_220809_bka_pan; Pos. 80

The main barriers to the rate of diffusion and dissemination were identified as inadequate supply of tablets—some of which were of inferior quality. Short battery life, poor touchscreen quality and low random-access memory sizes of 1 MB led to frequent crashes of the mobile app and data loss. A frontline user expressed disappointment in these devices:

I don’t know whether the tablets were donated to us or they were bought. I don’t know where they actually got them from. But I don’t think they are for this kind of technical jobs. Maybe they are for children.

GA_MT_LAB_14_QA_PT_220819_bka_pan; Pos. 99

The inconsistency of internet data bundle supply to users tested the motivation of some supervisors who took the initiative to buy internet data for their teams on some occasions. A supervisor expressed his fears about the risk of demotivation among his frontline users if this challenge persisted:

Here, if I don’t show interest, as an intrinsic motivation, that is that!! Because if I’ve used my data all this while, or if somebody says he doesn’t have data, that is it!! The person cannot access the SORMAS system, and you cannot blame the person unless you give the person data.

AS_MT_SUP_32_QA_PT_220710_bka_pan; Pos. 288

#### Outer Context

The outer context bordered on the prevailing national and international socioeconomic and political happenings that influenced any aspect of the implementation. Factors that facilitated the implementation included the financial and technical support from international partners, especially HZI and GIZ. Locally, the implementation benefited from the collaboration and technical support from the academia and public health training institutions, notably, the University of Ghana, Ghana Field Epidemiology and the Laboratory Training Program, Noguchi Memorial Institute for Medical Research, Kumasi Centre for Collaborative Research in Tropical Medicine, and Biomedical and Public Health Laboratory of the Council for Scientific and Industrial Research. The search for a national electronic tool for the COVID-19 pandemic response led to political involvement in the implementation of SORMAS. At a multidisciplinary national stakeholder evaluation of competing candidate electronic tools, SORMAS was adjudged the most ideal for both pandemic response and long-term public health surveillance and outbreak response. This was largely based on its compatibility with IDSR and adaptability and the satisfaction of GHS with its performance. The transparent evaluation and subsequent open political endorsement and directives from the level of the presidency for the nationwide deployment of SORMAS was cited as the most significant event that cut short its pilot in 2 regions for a switch to a nationwide scale-up, as narrated by one of the national public health officers:

So, we got to the presidency with several others [advocates of competing electronic tools] as I’ve mentioned, and then the meeting was chaired by the vice president himself. Then also present was the presidential advisor on health, and then other key people, and the vice president advisor on IT and digitalisation, yes, he was also there; and then some key other government people. So, at the beginning, I think [name of the person] was invited to brief them. So, we were all given opportunity to make a presentation, a 10-minute presentation each for our systems. And then based on that, then the decision as to what we were going to use nationally will be made.

IN_NA_EPI_52_QA_PT_220712_bka_pan; Pos. 68

Regarding the influence of political and business climate, they stated that partnering with private companies that depend largely on government contracts proved to be a risk. A change of government resulted in loss of contract and immediate closure of the first private partner. This in turn required the transfer of local software development and data hosting to another private IT company.

#### Linkage in Design and Implementation

This set of factors bordered on the existence and maintenance of a shared meaning and mission among public health actors for the adoption of SORMAS and how professional and human relations influenced the use of the system.

The study participants identified the prioritization of user-led design and development of task performance improvement requests as facilitators. Other named facilitators included the continual technical support from a broad participation of software developers (outside the SORMAS project) on GitHub discussions and collaborations and the existence of close and positive working relationship between developers and users. Good human and working relationships facilitated the implementation, as recounted by a participant:

In our particular case, it’s a very close working relationship between the users and the developers. And this is probably something...emmm, I don’t know. I’m not sure that this is happening everywhere but for sure, in Ghana, yes.

NA_GN_DV_61_QA_PT_ 220818_bka_pan; Pos. 51

And because we were working closely together, there weren’t hiccups. So, everybody knew themselves on the formal and informal level.

NA_GN_DV_61_QA_PT_220818_bka_pan; Pos. 57

However, they raised concerns about a risk of waning commitments in collaborations on skill and knowledge exchange between the local and international developers over time and how it could adversely affect sustainable use of the Ghana-specific SORMAS branch.

#### Prospects of Institutionalization

Regarding institutionalization, we assessed factors related to the sustained implementation and complete routinization of the use of SORMAS. These included the level of decentralization responsibilities of implementation shifted from the national to the regional and district levels. Key facilitators included the existence of a well-structured organogram with clear command and reporting lines—an all-inclusive management style, the modus operandi of which was collective brainstorming and consensus building. Other cited facilitators were the conduct of implementation review workshops and technical and financial support of major collaborators, namely, GIZ, HZI, WHO, US Centers for Disease Control and Prevention, and Nigeria Centre for Disease Control and Prevention.

The study participants identified factors that threatened the speed and eventual success of institutionalization that include a yet unestablished dedicated funding stream for eSurveillance, a substantial dependence on international partner funding, inadequate engagement of local government authorities and businesses for funding support, unevaluated maintenance cost of the system, and delays in transfer of data hosting expertise from the local IT partners to GHS. Another barrier was that some public health actors still perceive SORMAS as a project instead of an evolution of the surveillance system—a perception that could encourage retrogressive workforce attitudes and hinder efforts toward sustainability. When asked about the fate of SORMAS in Ghana beyond the support to implementation occasioned by the COVID-19 pandemic response, a study participant expressed the justification for and optimism mixed with anxiety about the prospects of institutionalization of SORMAS in Ghana:

Oooo emmm, since the tool is a surveillance and outbreak management and analysis system, any form of outbreak response, the tool is ready to accommodate that. Because, now looking at the outbreaks, emmm how do you call it...the epidemic-prone diseases that we have, and if on any of the days, tomorrow we wake up and then it is meningitis, SORMAS is there, if it is cholera, SORMAS is there. If any novel virus comes up like COVID-19 is doing now, SORMAS will really support. It is a matter of the developers incorporating the new disease into the system and it will be able to accommodate it. So, I’m sure it has come to stay. It has come to stay. Yes, it has come to stay. I’m praying that if today the developers in Germany say they are leaving it to Ghana and Nigeria, we will be able to get the skilled manpower to be able to handle the server so that it will be there and we can be able to continue to have a tool that can help us manage our surveillance system in the country.

NA_DS_ADM_51_QA_PT_220606_bka_pan; Pos. 38

## Discussion

### Principal Findings

We assessed the implementation of SORMAS in Ghana and identified facilitators, barriers, and sustainability strategies from the perspectives of public health actors actively involved in all aspects of the implementation.

The study identified 5 major strategic facilitators of the implementation of SORMAS in Ghana, namely, its design based on the IDSR workflow, adaptability to new tasks, well-established GHS organizational structure, good alignment with GHS medium-term and long-term goals for public health practice, and political endorsement. There were also operational facilitators including a strong national leadership, intrinsic motivation of frontline users, availability of in-country IT expertise, financial and technical support from inventors and international partners, demonstrated efficiency of SORMAS as both a surveillance and outbreak response process management tool, and relative advantages compared with existing systems for task performance (eg, real-time reporting, generation of case-base data, improved data quality, mobile offline capability, and integration of laboratory procedures).

Perhaps, the most important facilitator of the diffusion and dissemination of the system was the frontline users’ intrinsic motivation to use it. Consistent with the concept of balance of the “pains” and “gains” of adopting digital health innovations as espoused by Shaw et al [36], although some users were dissatisfied with delays in receiving internet data bundles, they took up the cost themselves—convinced that the benefits (gains) of using the system are worth their sacrifices (pains). Such levels of user altruism informed by their passion to use digital health innovations have also been reported from experiences of implementing mobile health tools in Ghana; Malawi [37]; Kenya [38]; Myanmar [39]; Brazil [40]; Los Angeles County, California [41]; Texas [42]; and the Netherlands [43]. However, this form of altruism is unsustainable, as aptly expressed by one of the frontline health workers:

Here, if I don’t show interest, as an intrinsic motivation, that is that!! Because if I’ve used my data all this while, or if somebody says he doesn’t have data, that is it!! The person cannot access the SORMAS system, and you cannot blame the person unless you give the person data.

AS_MT_SUP_32_QA_PT_220710_bka_pan; Pos. 288

The committed leadership of the health service managers and political endorsement at the level of the presidency translated to a boost in the commitment and confidence of the workforce. They had the assurance that their use of the system was appreciated by the highest office of the country. Users also considered themselves as being on a national assignment in the transition from paper-based surveillance and outbreak response to an electronic system. The positive user attitude derived from the high level of stewardship is consistent with user behaviors in the implementation of innovations in public health systems, as reported by Ginsburg et al [44] in their feasibility study of a digital innovation (mPneumonia) for pneumonia treatment in Ghana and an acceptability and usability study of a mobile health intervention for eye care in Kenya [45]. In addition, the all-inclusive management style of the GHS leadership on the implementation process, effective internal communication among supervisors and frontline users, trust of users in their leadership and the system, and field support by enthusiastic peer champions of SORMAS all contributed to sustaining the optimism for success in the face of operational challenges. Rightly so, this style of leadership and the support of champions have been recognized by digital health system implementers as important success conditions in Swaziland [46], Kenya [38], and Norway [47].

Major technical implementation barriers that our study identified include delays in completing the developments of technical interoperability between SORMAS and existing major data management systems, reliance on a private IT company for data hosting, and challenges with data synchronization. Operational barriers include the limited supply of quality data collection devices, substantial dependence on external funding, and unestablished estimates for full cost of implementation and medium-term to long-term maintenance.

One of the key benefits of adopting SORMAS is to improve the overall efficiency of task performance and reduce the workload. DHIS2 serves as the repository for all health data in Ghana, into which surveillance and outbreak data collected with SORMAS should be fed. However, the delays in achieving stable interoperability between these 2 systems undermine the expected efficiency of data transfer, as users are required to manually enter summary data already captured in SORMAS onto DHIS2. This challenge of system interoperability in the adoption of digital innovations in health care settings is rather common in both high-income countries and LMICs [9,42,43,48]. In the current case of Ghana, it poses a threat to a speedy completion of health system integration and institutionalization of SORMAS. A closely related barrier to long-term sustainability of the system is the yet inadequate technical expertise and infrastructural capacity of GHS to host the data collected with SORMAS. The availability of a private local partner to fill this gap is helpful, but its downside is that it deprives GHS of full control of its data. The external service also comes at an extra recurrent cost and competes for already stretched resources.

The challenge of synchronization undermines the real-time reporting and communication—the very properties of the system that make it a good management tool. Delayed or failed synchronizations were mostly a result of poor internet connectivity. Although the challenge of internet connectivity was more common in the rural districts, some of the study participants from urban districts experienced frequent episodes of poor connection. The challenge of reliable internet has improved over the years in LMICs but still poses a threat to the implementation of digital health tools in many settings, as corroborated by health worker experiences in Ghana [37,49], Kenya [38,45], Tanzania [50], Bangladesh [51], Myanmar [39], Brazil [40], India [52,53], and Ireland [54]. We recognize that the challenge of internet connectivity rests largely with the telecommunication industry in most countries and is not within the control of the health systems, even if they have unlimited funding to procure the best service subscriptions. Furthermore, the investments in national communication infrastructure are capital intensive, are consumer driven, and require good collaborations between the private and public sectors. In the past 2 decades, the leading internet providers in Ghana (Vodafone, Mobile Telecommunication Network, Expresso, Globacom, Airtel, and Tigo) have been largely privately owned and hence profit driven. As part of strategies to improve the penetration and quality of information and communications technology services, there have been calls for infrastructure sharing to contain the cost of investments and harness synergies among these service providers [55]. However, there have been challenges with mergers epitomized by the postmerger tensions between Airtel and Tigo that forced the government of Ghana to intervene and take over this merger [56]. This government takeover does not provide guarantee for better services because there is mixed evidence regarding the question of who provides more effective telecommunication services—the state or the private sector [57-59].

Cognizant of this multilayer of prerequisites for attaining the ideal quality of internet service vis-à-vis the ever-increasing need for real-time reporting in surveillance and outbreak response, the invention of Low-Bandwidth Database Synchronization by HZI offers a pragmatic solution, especially for LMICs [60]. Its implementation would overcome this challenge because it provides 3 alternate low-bandwidth options in 1 tool for data transmission and synchronization between mobile devices and central databases.

So far, given the level of technical and political stakeholder support, ongoing consolidation of system integration, and enthusiasm of frontline users, what remains as a serious threat to institutionalization and sustainability is a reliable national funding. There is good political support and appreciation of the utility of SORMAS. However, these have not translated to commensurate funding. The immediate reason is that the political support for the rapid nationwide scale-up of the implementation came owing to the need to respond to the COVID-19 pandemic. Thus, resources are unplanned and fragmented. A more systemic reason may simply be that health systems in LMICs, similar to most other sectors, are substantially dependent on external support, for which, new initiatives such as SORMAS do not become exceptions [61,62]. Although the rapid nationwide scale-up caused an increased demand on logistics, resulting in shortages, the continued support from the external partners, private sector, and government abated the logistics crisis over the course of the pandemic. This experience constitutes a reminder for governments and their national health institutes to maintain emergency funds and stockpiles as part of their emergency preparedness strategies [63-66]. Thus, to achieve this, our findings suggest 3 complimentary and mutually reinforcing approaches, namely, the commitment of central and local governments, broad national participation involving active financial and technical support from the private sector, and resource pooling from programs within GHS and among all health agencies in the country. Regarding the approaches to national ownership of health care financing in low-income countries, Kiendrébéogo and Meessen [67] also proposed a similar model, which they describe as “a journey with more than one pathway”—requiring broad stakeholder support including central governments, parliaments, health institutions, and the private sector. The private sector has also been identified as a key potential player in complementing the efforts of governments in health care financing in Nigeria [68,69]; Zimbabwe [70]; South Africa [71,72]; and more generally, for the African setting [73,74].

However, the timing of the introduction of SORMAS in Ghana could benefit from the national digitalization agenda that seeks to digitalize the operations of all public sectors, critical among which is health. This digitalization agenda is already receiving technical and funding support from in-country United Nations agencies and the World Bank for upgrading the overall national digital infrastructure [75-77]. We anticipate that the private telecommunication sector will remain a key player in realizing this goal, while also benefiting from a close public-private partnership. Furthermore, we anticipate that a joint stewardship of the United Nations, World Bank, private telecommunication sector, and government of Ghana could increase the chances of successful execution of the digitalization agenda. Ultimately, the envisaged penetration and quality of service of this agenda should minimize the challenges of internet connection in the use of SORMAS. A further boost to the sustainability outlook of SORMAS is Ghana’s formal adoption of eSurveillance consistent with WHO recommendations is expected to attract dedicated organizational budgetary support for its operationalization. Given that SORMAS is now the national electronic tool for surveillance, there is reason to be confident that its implementation will be sustained in the medium to long term. The ultimate institutionalization would benefit from the suggestions of users about galvanizing support from local government agencies and private business establishments at the lowest reporting levels.

We note that these stakeholder identified barriers and facilitators cut across all constructs of the model by Greenhalgh et al [32]. Thus, the outcome of the implementation is contingent on a dynamic nonlinear interaction of interdependent factors. This phenomenon has been widely recognized by other models and frameworks of implementation science [78], exemplified by the number and variety of barriers encountered in the implementation of a tuberculosis contact investigating system in Uganda [17] and the substantial gains of web-based disease monitoring and management system in the Netherlands [43]. The facilitators and barriers also cut nearly uniformly between urban and rural districts, except that internet connectivity was generally better in urban districts as would be expected. The uniformity of facilitators and barriers regarding implementation between the 2 study regions could be explained by 2 factors. First, the truncated piloting of the implementation in the Greater Accra Region did not allow for systematic evaluation and addressing of early bottlenecks. Second, the need for mass recruitment and equipping of new users across the country for the pandemic response posed similar challenges to the performance of SORMAS, workforce training, and related demands, all of which depend on a common resource pool.

From the foregoing, we infer that the facilitators and barriers identified by the study participants regarding the implementation of SORMAS in Ghana are not entirely unique to this tool and country. For example, just as its compatibility with IDSR would make it readily acceptable to countries of the WHO–Africa Region, so will the challenge of interoperability with the DHIS2 pose barriers to its integration with public health systems of these countries. The challenge of poor internet connectivity will confront the implementation of any digital tool in any country to the extent of the insufficiencies of the national telecommunication services—be it in the quality of bandwidth, geographical penetration, or other context-specific factors. Moreover, as in the case of Ghana, many LMICs receive financial support from international development partners, some of which are either short term or inconsistent [79]. Hence, the threat of implementation failure for lack of reliable and sustainable local funding should concern any such country that undertakes the institutionalization of digital systems for surveillance and outbreak response. The timing of adoption, organizational capacities and work culture, workforce skill sets, public-private partnerships, and overall political and business climate have relevance for the implementation of SORMAS in Ghana, as would be expected for the implementation of similar digital tools at scale in comparable settings. The interactions among these factors and how they influence the implementation are certain to vary to various extents depending on the specific contexts of adopting countries in normal times or during public health crises.

### Limitations and Strengths

Our study had 2 main limitations. First, apart from the 18% (4/22) of study participants who we purposively selected for their unique role in the implementation, the 82% (18/22) of other participants who responded promptly to our telephone calls were likely to be more interested in the system than the nonrespondents. Thus, it is possible that the range and magnitude of the barriers were underrated. However, their blunt expression of dissatisfaction about some aspects of the implementation suggests that they were balanced in their perspectives. In addition, we believe that our inclusion of all categories of stakeholders and independent observers served to cross-validate the responses of frontline workers, their supervisors, and software developers to reduce selection bias. Second, some resource persons declined the invitations to participate because they did not wish to have their interviews recorded. This possibly deprived the study of some more diverse perspectives. We minimized this risk by extensive probing during interviews and made follow-up calls to obtain complete data that were not immediately available or recallable or required consultation of coworkers or documents.

These limitations notwithstanding, our study had many strengths. First, the timing of the study allowed us to evaluate the implementation process in both normal and pandemic times. Thus, the richness of the findings has relevance for implementing similar tools in both scenarios. Second, the model by Greenhalgh et al [32] that we adapted as part of for our study design is a comprehensive model built from evidence obtained from an extensive systematic review of implementing innovations in health care organizations such as GHS. Thus, this model enabled us to identify a broad range of factors across all relevant aspects of the implementation, namely, technology, interactions of systems (internal, national, and international), and workforce behaviors. Third, our findings are also relevant in their timing as they could feed into evidence for funding prioritization for the adoption of eSurveillance as part of a national digitalization agenda. Fourth, our findings raise some important questions for further studies. An in-depth investigation of the business and political complexities of the telecommunication industry in Ghana could provide more insights about and possible solutions to the problems of poor information and communications technology services and how the private telecommunication industry could support public institutions in tackling the challenges of system interoperability that hamper the implementation and integration of digital systems. A review of funding models for the digitalization of surveillance in LMICs would also provide insights for adopting and adapting proven funding models to promote country ownership. As our findings reveal, the implementation of SORMAS in Ghana has so far benefited from a wide range of financial and technical contributions from the state, health workers, private sector, and international partners. Hence, a cost-benefit analysis would be a useful follow-up study to examine the direct, indirect, intangible, and opportunity costs of the implementation so far and could provide further insights for planning sustainable strategies.

We provide specific and targeted recommendations for the sustainable institutionalization of SORMAS in Ghana, which could also be useful for comparable LMICs (Table 2).

**Table 2:** Recommendations for the sustainable institutionalization of Surveillance Outbreak Response Management and Analysis System (SORMAS) in Ghana (2022).

Target stakeholder	Recommendations about sustainability strategies
Inventors	Standardize the data collection templates and data infrastructure to ensure multisystem compatibilities and data exchange among different health agencies Software developers should stabilize the interoperability between DHIS2^a^ and SORMAS to increase the efficiency of work
Implementing partners and collaborators	Consider a paradigm shift from the project perspective to national ownership and institutionalization of the system Consolidate the existing collaborations in knowledge, skill, and experience exchange among African and European software developers Establish a combined approach of maintaining and developing SORMAS software, in which GHS^b^ hosts their data and specifies epidemiological algorithms and the software developers perform the coding Maintain close interaction with the academic and research community to conduct studies for improving the features of SORMAS
Ghana government	On the basis of the national digitalization agenda of public institutions, the central government should consider providing additional funding support dedicated to eIDSR^c^ The government directive that established SORMAS as the national system for surveillance and pandemic response should be communicated to local governments in all 216 districts of the country to support implementation with logistics and funding, to support their reliance on the system for health data for local governance The members of parliament should allocate a percentage of their health insurance support to the district health directorates to purchase tablets and support the running costs of implementation
GHS	Managers should step up resource pooling of logistics, namely, tablets, laptops, desktops, Wi-Fi modems, and institutional Wi-Fi internet available from other programs to support implementation As part of resource pooling, managements of health facilities that generate internal revenue could support the procurement of tablets for their officers to support implementation The public health division of GHS could improve the consistency of internet data supply to users by engaging telecommunication companies to support with subsidized internet subscription packages The public health division should consider the alternative of subscribing to cloud services for hosting SORMAS data while they build the capacity for onsite server hosting as a backup Regional health directorates should plan and budget for biannual supportive supervision across their districts District health management teams should actively engage the local government and businesses to advocate for resource support for implementation The public health division should designate actionable SORMAS implementation focal individuals at the operational levels (national, regional, and district), as is practiced for other important programs Consolidate the user base by scaling downward to include subdistricts Institute regional refresher user trainings to keep up with system upgrades Promote the existing organizational culture of peer-to-peer training, especially for new recruits to the surveillance units of districts

^a^DHIS2: District Health Information System–version 2.

^b^GHS: Ghana Health Service.

^c^eIDSR: electronic Integrated Disease Surveillance and Response.

### Conclusions

The facilitators of and barriers to SORMAS implementation are multiple and interdependent. Important success conditions for implementation include enhanced scope and efficiency of task performance, strong technical and political stewardship, and self-motivated workforce. Inadequate funding, limited IT infrastructure, and lack of software development expertise are mutually reinforcing barriers to the progress of implementation and ownership. Some barriers are external, relate to overall national infrastructural development, and are not amenable even to unlimited project funding.

## Appendix

Multimedia Appendix 1 Coding system and intercoder reliability estimates.

Multimedia Appendix 2 Summary of key facilitators of and barriers to the implementation of Surveillance Outbreak Response Management and Analysis System in Ghana (2022).

## Abbreviations

DHIS2: District Health Information System–version 2
GHS: Ghana Health Service
GIZ: Deutsche gesellschaft für internationale Zusammenarbeit (German Corporation for International Cooperation)
HZI: Helmholtz-Zentrum für infektionsforschung (the Helmholtz Centre for Infection Research)
IDSR: Integrated Disease Surveillance and Response
LMICs: low- and middle-income countries
SORMAS: Surveillance Outbreak Response Management and Analysis System
WHO: World Health Organization

## Data Availability

The data collected for this study are not publicly available owing to data protection restrictions. However, they are available from the corresponding author upon reasonable request and subject to written clearance of the request by the data protection office of the Helmholtz Centre for Infection Research (*Helmholtz-Zentrum für infektionsforschung*)–Braunschweig.
