# Supplementary material for: Facilitators and Barriers in the Implementation of a Digital Surveillance and Outbreak Response System in Ghana Before and During the COVID-19 Pandemic: Qualitative Analysis of Stakeholder Interviews
Source: JMIR Form Res. 2023 Oct 20;7:e45715. doi: 10.2196/45715 (PMC10625076; doi:10.2196/45715)
Supplement: Multimedia Appendix 1 [file formative_v7i1e45715_app1.docx]

Multimedia Appendix 1: Coding system and inter-coder reliability estimates

| **Parent code** | **Sub-code** | **Code memo** | **%Crude Agreement** | **k (95% CI)** |
| --- | --- | --- | --- | --- |
| **Innovation** |  |  | 86.7 | 0.73 (0.60-0.86) |
|  | Task performance (relative advantage, risks and benefits) | Data that support a clear, unambiguous advantages in task performance, or otherwise |  |  |
|  | Knowledge requirements for use | Data that show whether or not the knowledge required to use the innovation can be codified and transferred from one context to another |  |  |
|  | Organisational compatibility | Data show whether or not the innovation is compatible with the intended adopter’s values, norms, and perceived needs, or otherwise |  |  |
|  | Reinvention and Trialability | Data that support the possibility to adapt, refine or modify an innovation to suit an adopter’s specific needs, or otherwise. |  |  |
|  | Technical support | Data on whether or not, the innovations is supported by training, help desk and customization support |  |  |
| **The Health System Antecedents and Readiness for the Innovation** |  |  | 84.6 | 0.69 (0.54-0.83) |
|  | Innovation-System fit | Data on whether or not the innovation fits the organisation’s existing values, norms, strategies, goals, skill mix, supporting technologies, and ways of working |  |  |
|  | Receptive context for change | Data supporting a strong leadership and a clear strategic vision, good managerial relations, risk-taking climate, clear goals, and priorities, or otherwise. |  |  |
|  | Dedicated time and resources | Data on whether or not the adopting organisation has a sufficient budget and adequate and slack resources to allocate to the implementation of the innovation |  |  |
|  | Absorptive capacity for new knowledge & skills | Data supporting pre-existing knowledge/skills base and the capacity to link innovation with its own existing knowledge base, or otherwise |  |  |
|  | Monitoring and Evaluation of implementation | Data on whether or not the existence of tight systems and appropriate skills to monitor and evaluate the impact of the innovation |  |  |
|  |  |  |  |  |
| **Adoption and Assimilation** |  |  | 92.9 | 0.86 (0.76-0.96) |
|  | Decision making | Data on the approach of decision making: Contingent? Collective? Authoritative? Other? |  |  |
|  | Evaluation for adoption | Data on whether or not users have good awareness of innovation, sufficient information about its use, clarity about personal and job implications e.g., cost, working hours etc. |  |  |
|  | Sustained efforts at implementation | Data illustrating practices aimed at consolidation of adoption at organisational level, or otherwise |  |  |
| **Diffusion and Dissemination** |  |  | 86.5 | 0.73 (0.65-0.81) |
|  | Workforce Training | Data on approaches of user trainings be they formal training workshops, on-the-job- peer to peer trainings, user personal efforts at gaining competence |  |  |
|  | Access to system (software and hardware) | Access to hardware (android devices, computers, barcode scanners); software (internet access to system and data servers for synchronisation) |  |  |
|  | Motivation | Data that illustrate individual and organisation motivation and extra efforts for the success of implementation |  |  |
|  | Trust | Data on perceptions of good will and support from the health system and political leaderships |  |  |
| **Linkage** |  |  | 80 | 0.62 (0.36-0.87) |
|  | Design approach | Data supporting a shared meaning and mission, effective knowledge transfer, user involvement in specification, and capture of user led-innovation, or otherwise |  |  |
|  | External change agents | Data supporting the existence of a system for team discussion and collaboration between users, developers, and other stakeholders to support continual use of innovation |  |  |
|  | Positive human relations | Deliberate actions/strategies to develop strong interpersonal relationships with existing and potential users and to explore and empathize with users' perspectives and experiences |  |  |
| **Outer Context** |  |  | 85.7 | 0.72 (0.53-0.91) |
|  | Incentives and mandates (political directives) | Data on whether or not there is governmental support including funding streams for the innovation |  |  |
|  | Political and business climate | Data depicting how general political happenings at institutional and national level influences the implementation of the innovation |  |  |
|  | Interorganisational norm-setting and networks | Data on comparable (homophilous) organizations within the sub-region or globally that have adopted the innovation or plan to do so |  |  |
| **Institutionalisation** |  |  | 87.5 | 0.75 (0.58-0.92) |
|  | Decentralisation | Data depicting whether or not there are adaptive/flexible structures and processes that support devolved decision making (e.g., strategic decision making devolved to departments, operational decision making devolved to teams on the ground |  |  |
|  | Internal communication | Data that depict whether or not there are effective communication mechanism and clear lines of command and responsibility in the adopting organisation |  |  |
|  | Human resource | Data on whether or not there is an early and widespread involvement of staff through formal facilitation initiatives to enhance their motivation, capacity, and competence |  |  |
|  | Inter-organisational networks and collaborations | Data illustrating whether or not there are any forms of support (financial, technical, etc.) from other public and private in country institutions or international organisations |  |  |
|  | Dedicated institutional/ national funding | Data on whether or not there exists a dedicated and reliable funding for implementation of innovation |  |  |
