# Supplementary material for: Facilitators and Barriers in the Implementation of a Digital Surveillance and Outbreak Response System in Ghana Before and During the COVID-19 Pandemic: Qualitative Analysis of Stakeholder Interviews
Source: JMIR Form Res. 2023 Oct 20;7:e45715. doi: 10.2196/45715 (PMC10625076; doi:10.2196/45715)
Supplement: Multimedia Appendix 2 [file formative_v7i1e45715_app2.docx]

**Multimedia Appendix 2.** Summary of key identified facilitators of and barriers to the implementation of Surveillance Outbreak Response Management and Analysis Systems (SORMAS) in Ghana (2022).

| Implementation domain | | Facilitators | Barriers |
| --- | --- | --- | --- |
| **Innovation** | | | |
|  | Task performance | - Many relative advantages - Real-time reporting - Case-base data - Improved data quality - Bidirectional communication - Offline mobile capability | - Interruptions of synchronization by poor internet connectivity |
|  | Knowledge requirements for use | - Smartphone savvy users^a^ | - Lack of expertise in server management |
|  | Organizational compatibility | - Compatibility with IDSR^b^—workflow and user roles | - User right restrictions |
|  | Technical support | - Use of GitHub as a supportive development platform - Availability of local IT expertise - The conduct of cyber penetration tests | - Delays in development and deployment of new features |
| **Adoption and assimilation** | | | |
|  | Decision-making | - Demonstrable fit with service needs - Extensive stakeholder engagements and all-inclusive decision-making - Open-source availability of the system - Political endorsement during the COVID-19 pandemic^c^ | - Uncertainties about sustainability |
|  | Evaluation for adoption | - Possibility for extension to OneHealth task modules - Integration of laboratory and surveillance workflows | - Concerns about interoperability with DHIS2^d^ |
|  | Sustained efforts at implementation | - Regular development and innovation meetings - Creating a Ghana-specific SORMAS branch^c^ - Participatory development on GitHub | - Multiplicity of electronic data systems^c^ - Additional cost of maintaining Ghana-specific branch^c^ |
| **Diffusion and dissemination** | | | |
|  | Workforce training | - Multiple approaches of training (workshops and peer-to-peer on the job) - Training of health managers | - Mass trainings in COVID-19 pandemic situation^c^ - Unscheduled refresher trainings^c^ |
|  | Access to system (software and hardware) | - Availability of backup power supply in some user institutions - Introduction of laboratory barcodes and scanners - Familiarity of laboratory staff with barcodes | - Unstable power supply - Poor internet service - Limited supply of hardware (computers, Android devices, barcodes, and barcode scanners)^c^ |
|  | Motivation | - Intrinsic motivation of users - Support of peer champions among users | —^e^ |
|  | Trust | - User trust in the utility of the system - User optimism in overcoming challenges over time | - Delays in the supply of internet data^c^ |
| **System antecedents and readiness** | | | |
|  | Innovation-system fit | - Opportune timing with adoption of e-IDSR | - Delayed implementation of interoperability with existing systems |
|  | Receptive context for change | - Risk taking and dedicated leadership - Intrinsic motivation of pioneer public national trainers - User enthusiasm for the tool | — |
|  | Dedicated time and resources | - Leadership’s prioritization of the system to invest a lot of time on implementation | - Limited supply of operational logistics^c^ - Substantial dependence on international partner funding |
|  | Absorptive capacity for new knowledge and skills | - Previous experience with other digital tools - Computer-literate surveillance officers | - Lack of infrastructure (local servers) and personnel expertise at GHS^f^ to host data |
|  | Monitoring and evaluation of implementation | - Planned monitoring and supervision during the pilot phase - Regular joint partner implementation review meetings | - Unscheduled supervisions based on urgent need^c^ - Inability to evaluate the pilot phase before national scale-up for COVID-19 response^c^ |
| **Outer context** | | | |
|  | Incentives and mandates (political directives) | - Advocacy support by top political hierarchy^c^ - Logistic and service support from central government^c^ | - Nonactive engagement of local governments and members of parliament |
|  | Political and business climate | - Ongoing national digitalization agenda | - Instability of business climate (political dependency) for private partners |
|  | Interorganizational norm setting and networks | - Financial support from international donors - Logistic support from local, private partners^c^ - Training and supervisory support from field epidemiology training program - User and system support from academic and research institutions | — |
| **Linkage** | | | |
|  | Shared meaning and mission | - User-led development of features - A team approach to data and cyber security | — |
|  | External change agents | - Open-source contributions to system development on GitHub - Continual technical support from the innovators | — |
|  | Positive human relations | - Established protocol of exchanges between developers and users - Good formal and informal team relations | — |
| **Institutionalization** | | | |
|  | Decentralization | - Use of SORMAS in all districts - Use of SORMAS by both research and private laboratories^c^ | - Inadequate decentralization of user rights to supervisors - Lack of SORMAS focal individuals at the district level - Incomplete decentralization to subdistricts |
|  | Internal communication | - Clear chain of command and reporting lines - Enhanced task communication and feedback - Conduct of internal implementation review meetings - Conduct of national implementation progress review workshop | - Inadequate resource pooling among GHS programs |
|  | Human resource | - Substantial computer literacy - Actively practicing workforce | - Shortage of competent trainers during rapid national scale-up^c^ - High attrition rate of recruits in the early phase of the pandemic^c^ |
|  | Interorganizational networks and collaborations | - Technical and financial support from international partners - Regional peer support from NCDC^g^ | — |
|  | Dedicated funding | — | - Lack of a dedicated national funding - Unestablished cost estimates for full implementation and maintenance |

^a^Users who are experienced in using several smartphone apps for work, study, entertainment, and communications and are also capable of basic troubleshooting.

^b^IDSR: Integrated Disease Surveillance and Response.

^c^Facilitators and barriers that were occasioned by the COVID-19 pandemic.

^d^DHIS2: District Health Information System–version 2.

^e^No identified corresponding facilitator or barrier for this domain

^f^GHS: Ghana Health Service.

^g^NCDC: Nigeria Centre for Disease Control and Prevention.
